# Supplementary material for: Electroacupuncture for acute postoperative pain during coughing after video-assisted thoracoscopic surgery: study protocol for a pilot randomized controlled trial
Source: PLoS One. 2025 Mar 26;20(3):e0316698. doi: 10.1371/journal.pone.0316698 (PMC11940426; doi:10.1371/journal.pone.0316698)
Supplement: S2 File — (PDF) [file pone.0316698.s002.pdf]

Trial Protocol

Study name: Electroacupuncture for acute postoperative pain after video-assisted thoracoscopic surgery: A Randomized Controlled Trial

Project leader: Cun-Zhi Liu

Undertaker: Beijing University of Chinese Medicine

Contacts: Dan-Tong Zhang

Background

Video-assisted thoracoscopic surgery (VATS) has become the primary surgical approach in thoracic surgery. Compared with thoracotomy, VATS is notable for less trauma, lower postoperative pain levels, better quality of life shorter length of hospitalization, and lower complication rates. Despite numerous analgesic strategies in clinical practice, including thoracic epidural analgesia, paravertebral block, and patient-controlled analgesia, acute postoperative pain still exists after VATS.

Acute postoperative pain is closely related to operation time, chest tube duration, and postoperative activities. After surgery, doctors will encourage patients to cough to promote the expulsion of respiratory secretions, but patients may be afraid to cough forcefully due to postoperative acute pain. About 59% of patients experienced moderate to severe acute pain during the first three days after VATS. In contrast, postoperative pain when coughing is often more severe than when resting. Approximately 51.7% of patients experienced moderate to severe pain when coughing within the first 24 hours after surgery. On postoperative day 7, around 20% of patients were still encountering moderate and severe pain while coughing.

Notably, inadequate management of acute postoperative pain frequently results in chronic post-surgical pain (CPSP). About 43.99% of patients reported CPSP after VATS, and the incidence of moderate or severe chronic

pain is 14.71% among them. CPSP will reduce the quality of life of patients and affect their physical and mental health. The efficacy of drug treatment is limited for CPSP at present. Acute postoperative pain control and prevention of CPSP still lack effective interventions.

Due to its significant analgesic effect, electroacupuncture (EA) has been widely used to alleviate various clinical pains such as knee osteoarthritis, acute renal colic, and low back pain. A previous clinical trial on managing postoperative pain through acupuncture following thoracoscopic pulmonary resection found that the numerical rating scale (NRS) scores at 24h, 48h, and 3 months after surgery in the EA group were lower than those in the sham acupuncture group. The total pressing numbers of patient-controlled intravenous analgesia (PCIA) also showed the same result. EA might be efficacious in alleviating acute postoperative pain following VATS, but many shortcomings in existing clinical studies, such as small amount of research, small sample size, low research quality and so on. Therefore, we designed this randomized controlled trial to observe whether EA combined with standard care can alleviate the acute pain of patients with non-small cell lung cancer (NSCLC) after VATS compared with sham electroacupuncture (SA).

Objectives

This trial aims to assess the feasibility and efficacy of EA with standard care versus SA with standard care for acute postoperative pain after VATS.

Hypothesis testing:

H0: Efficacy of EA = Efficacy of SA

H1: Efficacy of EA ≠ Efficacy of SA

Study design

This trial is a multi-center, randomized, sham controlled acupuncture clinical trial that follows the CONSORT and the STRICTA. Fig 1 shows the flow diagram of the trial. Fig 2 shows the visiting time point.

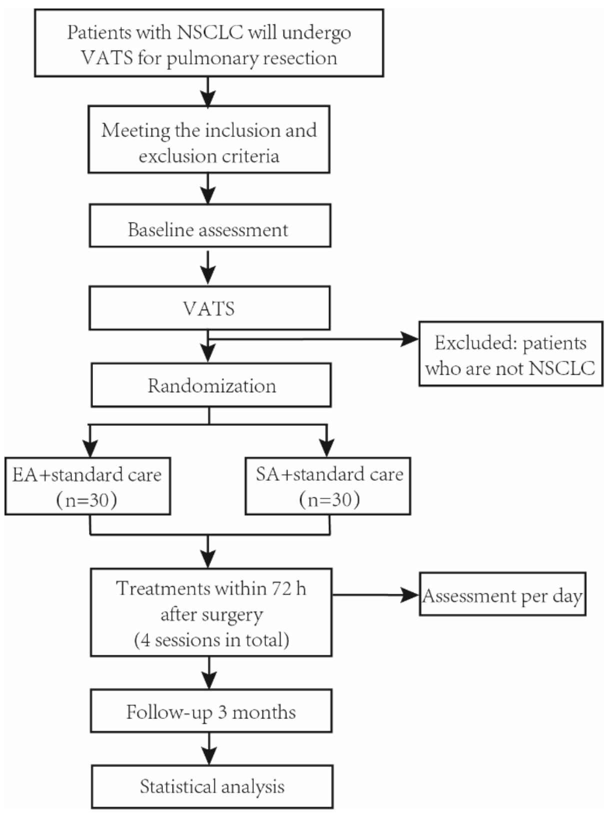

Fig 1. Study flow diagram.

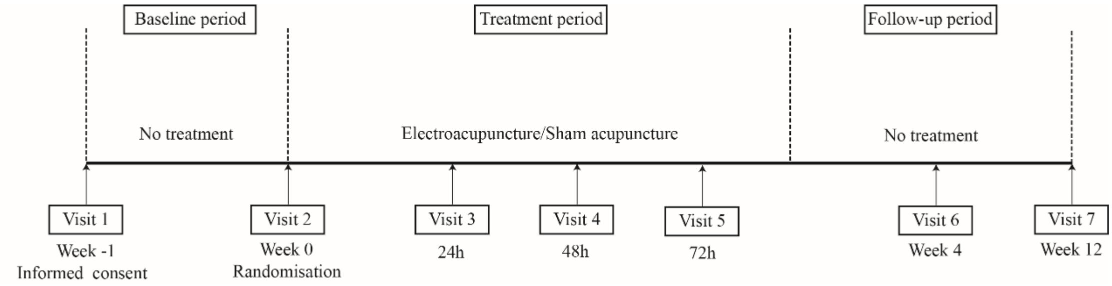

Fig 2. Visiting time point.

Participants

The participants are patients with NSCLC who undergo VATS in two hospitals. The two hospitals are as follows:

① The First Affiliated Hospital of Zhengzhou University

② Cancer Hospital Chinese Academy of Medical Sciences

Participants will be recruited through wards.

Diagnostic criteria: Patients diagnosed as NSCLC by pathology.

Inclusion criteria:

1. Men or women aged 18 to 75 years

2. Patients diagnosed with NSCLC before or during surgery

3. Undergoing VATS for pulmonary resection (including lung wedge resection, segmentectomy, and pulmonary lobectomy) for the first time

4. American Society of Anesthesiologists physical status I or II 筛选表加

5. Having no communication barriers and can cooperate with 3 months telephone follow-up

6. Willing to sign informed consent

Exclusion criteria:

1. Patients with a history of previous chest surgery (e.g., mastectomy, thoracotomy, or thoracoscopic surgery), or chronic pain
2. Patients with a history of alcohol abuse or drug misuse
3. Patients with a history of preoperative respiratory infection, chronic cough, sputum production, or postnasal drip syndrome
4. Having received chemotherapy, radiation therapy, or acupuncture treatment within 3 months prior to the trial
5. Conversion to open surgery or require postoperative admission to the Intensive Care Unit
6. Patients who are allergic to analgesic drugs and unwilling to use patient-controlled intravenous analgesia (PCIA) after surgery
7. Planning to receive additional surgeries within 3 months postoperatively
8. Having neurological illnesses, pregnancy, lactation, severe hypertension, diabetes, cardiovascular disease, serious hepatic or renal insufficiency, or infectious diseases
9. Having metal allergy, coagulation abnormalities, infections at the selected acupoint sites, or the presence of implantable medical devices such as pacemakers
10. Participating in other clinical trials

Those who meet one or more of the above requirements will be excluded.

Randomization

Stratified block randomization will be applied with stratification according to the two research centers and variable block lengths. Sixty eligible patients will be randomly assigned to the EA or SA group in a 1:1 ratio. A statistician not involved in the implementation or statistical analysis of the trial will generate the random sequences using the STATA statistical software (version 12.0; StataCorp LP). The random number is kept by the independent administrator, who does not participate in the trial intervention, evaluation, and statistics. The CRC will contact an independent administrator by phone to obtain randomization information.

Blinding

Patients, outcome assessors, and the statistician will be blinded to group assignments to reduce potential bias. The acupuncturist is responsible for the acupuncture operation and using an EA apparatus. The appearance of the EA apparatus in the two groups will be identical, but the internal wires of the EA apparatus in the SA group will be cut to inhibit normal operation. Due to the characteristics of the acupuncture, acupuncturists will not be masked.

Procedure

1. conventional therapy

All the patients will be scheduled for surgery in the morning and will undergo general anesthesia during the surgery. All patients will receive PCIA for postoperative pain management. The analgesic solution, containing 14 mg of hydromorphone, 0.15 mg of palonosetron, and 100 mg of flurbiprofen axetil, will be diluted with normal saline to 200 mL (background dose, 3 mL h^{-1}; bolus, 4mL; and interval, 15 min). If the numeric rating scale (NRS) pain score at rest is ≥4 after 2 consecutive PCIA, additional dezocine will be administered intravenously as rescue medication (Intravenous injection, 5 mg/ time, no more than 120 mg/ day at most, and the interval between two doses is not less than 2 h).

2. Interventions

Patients will receive four sessions of EA or SA within 72 h after surgery (treatment timepoint 1: upon patients return to the ward, treatment timepoint 2: 6 h after surgery, treatment timepoint 3: at 2 pm on postoperative day 1, and treatment timepoint 4: at 2 pm on postoperative day 2). Each treatment will continue for 30 minutes.

(1) Electroacupuncture group

Acupoints: the bilateral *Taichong* (LR3), *Yanglingquan* (GB34), *Kongzui* (LU6), *Neiguan* (PC6), *Hegu*(LI4), and *Neimadian* (Extra point).(Table 1)

Needles: Single-use sterile acupuncture needles with 0.25 mm × 40 mm

and 0.40 mm × 50 mm.

Operation: After the acupuncture site and the acupuncturist's hand are strictly disinfected with 75% alcohol, the acupuncturist holds needles of in his right hand. LR3, GB34, LU6, PC6, and LI4 will be inserted with needles of 0.25 mm × 40 mm. *Neimadian* will be inserted with needles of 0.40 mm × 50 mm. There is manipulation for *de qi* (*Neimadian*: the first toe dorsiflexion, and other toes flexion, and other acupoints have a sensation combining sourness, numbness, distention, and heaviness) at each acupoint. Electric stimulation will be used for 30 minutes with an EA apparatus. The paired electrodes of the EA apparatus will be attached to the needle handles at GB34 and *Neimadian*. Continuous waves at a frequency of 2 Hz will be set, gradually increasing the electric current to a tolerable level for the patient.

Table 1 Location of acupoints for EA group

**Acupoints**	**Locations**[a]	**Angle**	**Depth**
Taichong (LR3)	On the dorsum of the foot, between the first and second metatarsal bones, in the depression distal to the junction of the bases of the two bones, over the dorsalis	straight insert	0.5-1 cun

	pedis artery		
Yanglingquan (GB34)	On the fibular aspect of the leg, in the depression anterior and distal to the head of the fibula		1-1.5 cun
Kongzui (LU6)	On the anterolateral aspect of the forearm, on the line connecting LU5 with LU9, 7 cun superior to the palmar wrist crease		0.5-1 cun
Neiguan (PC6)	On the anterior aspect of the forearm, between the tendons of the palmaris longus and the flexor carpi radialis, 2 cun proximal to the palmar wrist crease		0.5-1 cun
Hegu (LI4)	On the dorsum of the hand, radial to the midpoint of the second metacarpal bone		0.5-1 cun
Neimadian (Extra point)	On the medial side of the lower leg, 7 cun above the medial malleolus and 0.5 cun		0.5-1 cun

	from post edge of the tibia		

(2) Sham electroacupuncture group

Acupoints: Six non-acupoints.

Needles: Single-use sterile acupuncture needles with 0.25 mm × 25 mm and 0.40 mm × 25 mm.

Operation: After the acupuncture site and the acupuncturist's hands are strictly disinfected with 75% alcohol. The needling depth will be 2-3 mm. There will be no manipulation to reach *de qi* at any non-acupoint. The appearance of the EA apparatus will be identical in both groups. However, the internal wires of the EA apparatus will be cut in the SA group. Hence, no electric current will be delivered while the apparatus will be turned on. Electric stimulation will be used for 30 minutes with an EA apparatus.

Table 2 Location of non-acupoints for SA group

**Non-Acupoints**	**Locations**
Non-Acupoint 1	2 cun above the medial malleolus (between the liver and spleen meridian)
Non-Acupoint 2	In the middle of *Yanglingquan* (GB34) and *Zusanli* (ST36) points (between the gallbladder and stomach meridian)
Non-Acupoint 3	on the ulnar side of the arm, half way between the epicondylus medialis of the humerus and the

	ulnar side of the wrist
Non-Acupoint 4	Half way between the tip of the elbow and the axilla
Non-Acupoint 5	On the front arm of deltoid muscle and biceps brachi junction
Non-Acupoint 6	2 cun above Sanyinjiao (SP6), between the liver and spleen meridian)

If the subjects take drugs unrelated to analgesia, it is also necessary to record the reasons for taking drugs, the name of the drugs, the mode and dosage of drugs, and the time of taking drugs and stopping drugs.

Outcomes

Primary outcome

The average postoperative pain score during coughing is recorded using NRS within 72 h after surgery.

NRS scale is an effective tool to evaluate pain, with high reliability and validity, easy to record, and widely used. The scale requires patients to rate themselves according to the degree on a 10-point scale, which is divided into 1-10 grades. According to the corresponding figures, the pain can be divided into different degrees, and the middle from 0-10 represents the pain of different degrees that gradually increases. 0 is no pain, 1-3 is mild pain, 4-6 is moderate pain, and 7-10 is severe pain.

Secondary outcomes

(1) The average pain score during rest, coughing, and mobilization (moving from the lying to the sitting position) will be measured by NRS (assessment time: 24h, 48h, and 72h after VATS).

(2) Pain scores during rest and the incidence of CPSP (defined as NRS ≥ 1) at month 3 after surgery.

(3) Five-level EuroQol five-dimensional questionnaire (EQ-5D-5L) (assessment time: before surgery and at 24h, 72h, and month 3 after surgery).

(4) The severity of postoperative cough measured by cough symptom score (assessment time: month 1 and month 3 after surgery).

(5) The dosage of postoperative analgesic drugs and rescue medication. (assessment time: the whole study period).

(6) The postoperative pulmonary complications (atelectasis, pulmonary infection, pleural effusion, pneumothorax, etc.) and adverse events of acupuncture and drugs (assessment time: the whole study period).

(7) First expectorated sputum (assessment time: the whole study period).

(8) Chest tube duration (assessment time: the whole study period).

(9) Length of postoperative hospital stay (assessment time: the whole study period).

(10) Blinding assessment (assessment time: after the second treatment).

Feasibility indicators

Feasibility indicators include patient recruitment rate, randomization rate, treatment compliance, and subject retention rate. Compliance refers to the proportion of acupuncture courses obtained from the expected course of treatment.

Sample size calculation

Based on previous studies, the average postoperative pain score during coughing for the EA and SA groups is estimated to be 3.0 ± 1.7 and 4.4 ± 1.7. To achieve a statistical power of 80% with a two-sided significance level of 5%, 24 patients per group are required. Sixty patients (30 per group) are required, accounting for a dropout rate of 20%. This pilot trial is intended to establish initial data for the primary outcome measure, and the results will be used to calculate the sample size for the next larger randomized controlled trial.

Statistical analysis

Statistical analysis will be processed using SPSS Statistics 27. Measurement data will be described as mean ± standard deviations (M±SD), median, or interquartile range. Categorical data will be presented by frequency, percentage, or constituent ratio. Independent t-test or

Wilcoxon rank-sum test will be used to compare the measurement data between groups. The chi-squared test or Wilcoxon rank-sum test will be used to compare the categorical data between groups. The significant level is $P < 0.05$, which is considered to be statistically significant.

All randomly assigned patients will be included in the intention-to-treat (ITT) analysis. The per-protocol (PP) analysis, which includes participants completing 80% of the treatment without major protocol deviations or omissions will be used for the primary outcome as sensitivity analysis.

Normally distributed continuous variables will be analyzed using independent t-test. Skewed continuous variables will be analyzed using Wilcoxon rank-sum test. Categorical data will be analyzed using the chi-squared test or Wilcoxon rank-sum test. We will perform a predefined subgroup analysis on primary endpoints according to the chest tube duration, the number of incisions, etc.

Drop-out case and handling

Drop-out criteria: although the subjects through informed consent and obtained the random number by qualified screening, they could not complete the course of treatment and observation period stipulated in the study protocol, which was regarded as a drop-out case.

Handling of drop-out cases: When the subjects drop out, the researcher

should contact the subjects as much as possible by visiting the house, making an appointment for follow-up, telephone, and letter, asking for reasons, recording the last acupuncture time, and completing the evaluation items that can be completed. The relevant study data of drop-out cases should be properly preserved, which is not only for keeping, but also for ITT analysis. Patients who drop out do not need to be recruited.

Withdraw criteria

The subject proposed to withdraw:

① Poor curative effect;

② Can't tolerate adverse events;

③ Hope to take other treatment methods;

④ Withdraw voluntarily without any reason.

Termination standard

① The patient has serious adverse events, and the clinical trial of this case needs to be stopped according to the doctor's judgment.

② If other diseases affect the observation of the trial, the clinical trial should be stopped according to the doctor's judgment, and the case should be treated as invalid.

③ There are important deviations in the implementation of the clinical study protocol, such as poor compliance, which makes it difficult to

evaluate the efficacy of acupuncture.

④ The subject is unwilling to continue the clinical trial during the clinical trial and asks the competent doctor to withdraw from the clinical trial.

Elimination criteria

① Subjects entered the trial in violation of inclusion criteria and exclusion criteria.

② Combined medication in violation of the study protocol.

③ Wrong treatment grouping.

④ The subjects' medication compliance is very poor.

Quality control

① Subjects will be included in strict accordance with the criteria of diagnosis, inclusion, and exclusion.

② The random number is managed by the independent administrator. The random number is given only after obtaining the specific information of qualified subjects and recording it, so as to ensure the allocation concealment and prevent the random number from leaking or drifting in advance.

③ After discussion by experts, the relevant standard operating procedures are formulated for each step of the experiment, so that there is a unified standard for the operation of each step and a basis to follow when

opinions differ.

④ Researchers must undergo unified training. The contents include being familiar with the objectives and requirements of this study, mastering the relevant diagnosis and treatment standards, random distribution methods, acupuncture operation methods, and the use of evaluation forms, and setting different training periods for researchers with different divisions. Researchers with the same division of labor in the research center need to pass the consistency test to carry out the experiment, and the consistency test is required to use video materials for film preservation.

⑤ The research sub-center inspectors monitor completed cases twice a month, identify the existing problems, and guide them to correct.

⑥ The inspectors of the research center review the study process and examine the ethical issues involved in the study. Order them to rectify if they do not comply with the ethical principles. Regularly and irregularly monitor the study process, point out the existing problems and guide them to correct.

Risk and Management

Acupuncture Risk

Evaluation of adverse events at any time in the process of acupuncture includes hematoma, fainting, continuous post-needling pain, etc.

Treatment measures for fainting needle

Stop acupuncture, pull all the needles out, and ask the patient to prostrate and keep warm.

Treatment measures for hematoma

When a small piece of bruise is caused by a small amount of subcutaneous bleeding, it generally does not need to be treated, and it can subside on its own. When the local swelling and pain are severe and the bruised area is large, a cold compress should be used to stop bleeding.

Treatment measures for continuous post-needling pain

If the severity of continuous post-needling pain is mild, it can disappear or improve by local upward and downward patrol with fingers. In addition to local upward and downward patrol, moxibustion with moxa sticks can also be used for severe continuous post-needling pain, which can also be quickly eliminated.

Summary and Data Preservation

After the clinical observation is completed, the project management office is responsible for summarizing the data. All the original materials are reviewed and signed by the person in charge of the sub-center. The scientific research department will seal it and keep it in the scientific research data archives of the project undertaking unit.
