# Supplementary material for: Electroacupuncture for acute postoperative pain during coughing after video-assisted thoracoscopic surgery: study protocol for a pilot randomized controlled trial
Source: PLoS One. 2025 Mar 26;20(3):e0316698. doi: 10.1371/journal.pone.0316698 (PMC11940426; doi:10.1371/journal.pone.0316698)
Supplement: S3 File — (PDF) [file pone.0316698.s003.pdf]

试　验　方　案
（Trial Protocol）

试验名称： 电针治疗胸腔镜术后急性疼痛的随机对照临床试验

课题负责人：刘存志

承担单位： 北京中医药大学

联 系 人：张丹桐

研究背景

视频辅助胸腔镜手术(Video-Assisted Thoracoscopic Surgery，VATS)因其手术伤口比传统的开胸手术更小、术后疼痛程度更低、恢复更快等优点，现已成为胸外科主要的手术方式。尽管临床实践中有许多疼痛管理措施，包括硬膜外镇痛、椎旁神经阻滞、静脉镇痛等，但胸腔镜术后急性疼痛仍然存在。

术后急性疼痛与手术时间、放置引流管、术后活动等因素密切相关。医生在术后会鼓励患者咳嗽，以促进呼吸道分泌物的咳出，而患者会因为术后急性疼痛不敢用力咳嗽。在 VATS 术后前三天，约有 59%的患者会出现中度至重度急性疼痛。术后患者咳嗽时的疼痛常较休息时更为剧烈。约有 51.7%的患者在术后 24 小时咳嗽时会出现中重度疼痛，20%的患者在术后第 7 天咳嗽时仍存在中重度疼痛。术后急性疼痛控制不佳往往会导致术后慢性疼痛（Chronic Post-Surgical Pain，CPSP）。VATS 术后 CPSP 的发病率约为 43.99%，其中中重度 CPSP 的发病率为 14.71%。CPSP 会对患者生活质量产生负面影响，且目前药物治疗的疗效有限，仍需有效干预措施控制术后急性疼痛和预防术后慢性疼痛。

电针因其镇痛作用显著，现已被广泛运用于缓解各种临床疼痛，如膝骨关节炎、肾绞痛、腰痛等。既往观察电针对胸腔镜手术患者术后疼痛影响的研究（n=86），发现电针组术后 24h、48h、3 个月的数字评定量表(Numerical Rating Scale，NRS)疼痛强度评分和患者静脉自控镇痛(Patient-controlled Intravenous Analgesia，PCIA)按压总次数均低于假针组。电针可能可以有效缓解胸腔镜术后患者的疼痛，但现有临床研究数量少，样本量小，研究质量较低。这项随机临床试验将观察与假电针相比，电针联合常规治疗能否减轻非小细胞肺癌患者胸腔镜肺切除术后急性疼痛程度。

研究目的

探讨与假电针相比，电针联合常规疗法治疗胸腔镜术后急性疼痛的可行性和有效性。

假设检验：

H0：电针疗效＝假针疗效

H1：电针疗效≠假针疗效

试验类型

本试验是遵循CONSORT声明和STRICTA声明的多中心、随机、安慰对照针刺临床试验。试验流程见图 1、图 2。

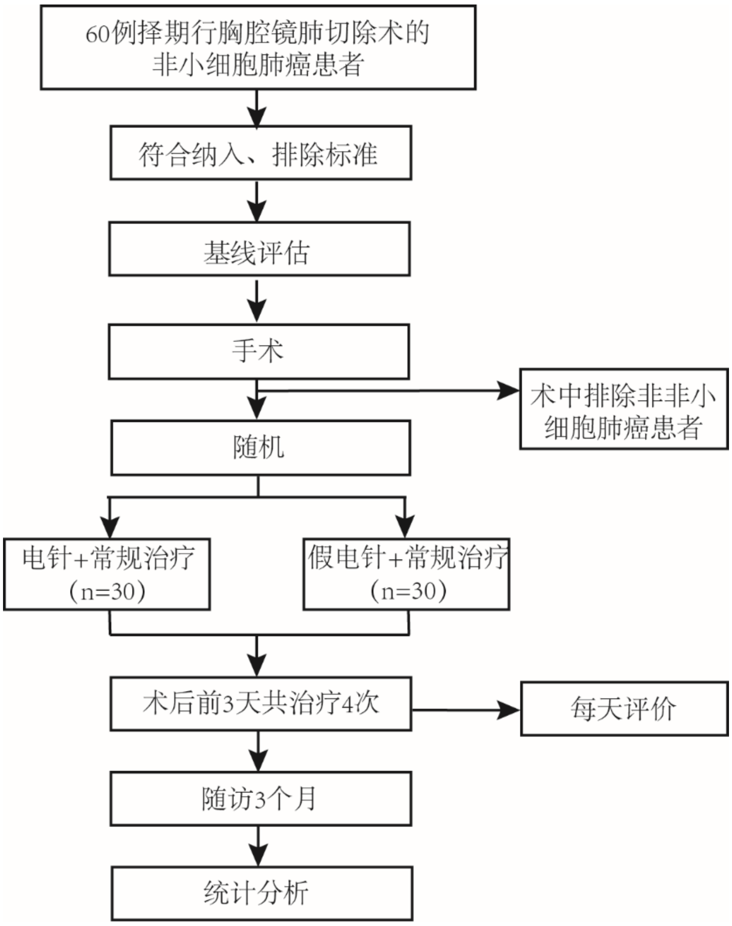

图 1 流程图

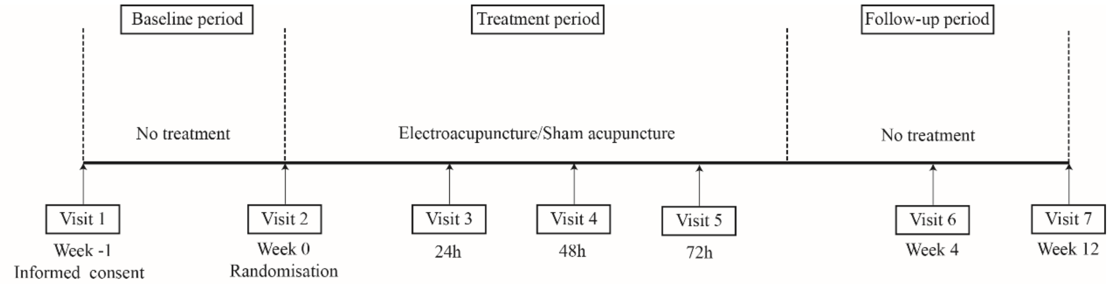

图 2 访视图

试验对象

受试对象来自于 2 家医院的择期行胸腔镜肺切除术的非小细胞肺癌患者。具体医院如下：

① 郑州大学第一附属医院；

② 中国医学科学院肿瘤医院；

受试对象通过病房招募。

诊断标准：

通过病理学诊断，明确为非小细胞肺癌的患者

纳入标准：

①年龄 18-75 岁，男女不限。

②术前或术中诊断为非小细胞肺癌。

③首次行胸腔镜肺切除术（包括肺楔形切除术、肺段切除术、肺叶切除术）。

④美国麻醉医师协会 ASA 身体状况分级Ⅰ或Ⅱ。

⑤无沟通障碍，可以配合 3 个月的电话随访。

⑥签署知情同意书。

排除标准：

①既往有胸部手术史（包括但不限于乳腺切除术、剖胸术、胸腔镜手术）、以及慢性疼痛史。

②酗酒或药物滥用史。

③术前有呼吸道感染及慢性咳嗽咳痰史，或鼻后滴漏综合征

④接受过化疗或放疗诱导治疗，或在过去 3 个月内接受过针灸。

⑤术中转为开胸手术，或术后进入 ICU 继续治疗者。

⑥对镇痛药物过敏，术后不愿意使用静脉自控镇痛者。

⑦在术后 3 个月内有其它手术计划。

⑧有神经系统疾病、妊娠、哺乳期、严重高血压、糖尿病、心血管疾病、严重肝肾疾病和传染性疾病。

⑨金属过敏、凝血功能障碍和针刺部位感染，有心脏起搏器等植入性医疗器械。

⑩同时参加其他临床研究。

满足以上 1 项或多项要求者即予排除

随机

试验采用中央分层区组随机法，以中心为分层因素，区组长度不固定，将60 例受试者按 1:1 的比例随机分配至电针组、假针组。由不参与试验的统计人员采用STATA软件产生随机号码，随机号码由随机号管理员保管，其不参与试验干预、评价及统计。当有合格的受试者时，临床研究协调员(Clinical research coordinator，CRC)通过与随机号管理员联系后获取随机号，实现分配隐藏及中央随机。

盲法

对受试者、结局评价者、数据统计分析的研究人员隐藏组别信息。针灸医师负责针灸操作并连接电针仪，假针组所连接的电针仪中连接电线内部断开，外表与电针组电针仪一致。由于针灸本身操作特性，患者的组别信息无法对针灸医师设盲。

干预

1.常规治疗

安排每位患者在上午进行手术，2 组术中均采用全身麻醉，术后接患者静脉自控镇痛(PCIA)。PCIA 设计：氢吗啡酮 14 mg+帕洛诺司琼 0.15 mg+氟比洛芬酯 100 mg+生理盐水=200 mL，持续输液量为 3 mL/h，自控给药量 4 mL，锁定时间 15 min，若 PCIA 泵按压两次无效且患者 NRS>4 分，则给予地佐辛（静脉注射，5 mg/次，最多不超过 120 mg/天，两次用药间隔不少于 2 h）进行补救镇痛。

2.干预措施

电针和假针治疗在患者返回病房后即刻、术后 6 h、术后第一天 14:00 和术后第二天 14:00 进行，每次治疗留针 30 min，术后前 3 天共计治疗 4 次。

（1）电针组

取穴：双侧太冲、阳陵泉、孔最、内关、合谷、内麻点（表 1）

针具：一次性无菌针灸针（0.25mm×40mm 和 0.40mm×50 mm）

操作：在针刺部位及针灸医师手部用 75%酒精严格消毒后，针灸师右手持 0.25mm×40mm 一次性针灸针进行针刺操作(内麻点使用 0.40mm×50mm 一次性针灸针)，平补平泻，针刺得气(内麻点：四趾屈曲、母趾背伸；其余穴位：有酸麻胀重感)后，电针连接内麻点—阳陵泉（2 对电极），采用连续波，

频率(2Hz)，电流逐渐增大以患者能耐受为宜，刺激 30min。

表 1 腧穴定位

穴位名称	定位	针刺角度	针刺深度
太冲	在足背，第 1、2 跖骨间，跖骨底结合部前方凹陷中，或触及动脉搏动	直刺	0.5-1 寸
阳陵泉	在小腿外侧，腓骨头前下方凹陷中		1-1.5 寸
孔最	在前臂前外侧，腕掌侧远端横纹上 7 寸，尺泽与太渊连线上		0.5-1 寸
内关	在前臂前侧，腕掌侧远端横纹上 2 寸，掌长肌腱与桡侧腕屈肌腱之间		0.5-1 寸
合谷	位于手背，在第 1、2 掌骨间，第 2 掌骨桡侧的中点		0.5-1 寸
内麻点	在小腿内侧，内踝上 7 寸，胫骨后缘约 0.5 寸处		1-1.5 寸

（2）假针组

取穴：6 个非穴（表 2）

针具：一次性无菌针灸针（0.25mm×25mm 和 0.40mm×25 mm）

操作：在针刺部位及针灸医师手部用 75%酒精严格消毒后，针灸医师使用一次性无菌针灸针浅刺皮下约 2-3mm，不进行手法操作，不得气，连接电针，电针仪不通电（中间电线剪断，外表如常，电针仪显示接通状态，实际未通电），留针 30min。

表 2 非穴定位

穴位名称	定位
非穴点 1	在小腿内侧，内踝上 2 寸，胫骨内侧面正中
非穴点 2	在小腿外侧，阳陵泉和足三里中间
非穴点 3	在前臂内侧，肱骨内上髁与尺骨腕部尺侧缘的连线中点处
非穴点 4	在肘内侧，肘尖与腋窝连线中点
非穴点 5	在上臂内侧，臂内前缘三角肌和肱二头肌交界处
非穴点 6	在小腿内侧，胫骨与腓骨之间，三阴交向上 2 寸处

如受试者服用与镇痛不相关的药物，也需要记录受试者服用药物原因，用药

名称，用药方式及剂量，服药及停药时间。

结局测量

主要指标

用 NRS 量表测评术后 72 小时内咳嗽时的平均疼痛程度

NRS 量表是评估疼痛的有效工具，具有较高信度与效度，易于记录，应用广泛。该量表需要患者在 10 分制的标尺上根据程度进行自我评级，共分为 1-10 级，根据对应数字可以将疼痛分为不同程度，中间从 0-10 代表逐渐加重的不同程度的疼痛，即 0 级为没有疼痛，1-3 级为轻度疼痛，4-6 级为中度疼痛，7-10 级为重度疼痛。

次要指标

（1）每日咳嗽时、安静时、活动时（从躺姿变为坐姿）平均 NRS 疼痛评分（评价时间点：术后 24 h、48 h、72 h）

（2）术后第 3 个月的疼痛程度和慢性疼痛发病率（定义：NRS>1）

（3）五水平五维健康量表（EQ-5D-5L）（评价时间点：基线、24 h、72 h、3 个月）

（4）咳嗽症状积分表测得的术后咳嗽严重程度（评价时间点：1 个月、3 个月）

（5）术后镇痛药物用量、应急药物用量（评价时间点：整个研究期间）

（6）肺部并发症（肺不张、肺部感染、胸腔积液、气胸等）、针刺和药物的不良反应（评价时间点：整个研究期间）

（7）第一次排痰时间（评价时间点：整个研究期间）

（8）胸腔引流管放置时间（评价时间点：整个研究期间）

（9）住院时间（评价时间点：整个研究期间）

（10）盲法评价（评价时间点：第 2 次治疗后）

可行性指标

可行性指标包括患者招募率、随机化率、治疗依从性和受试者保留率。依从性指从预期疗程中获得的针灸疗程的比例。

样本量估算

基于前期研究，我们预计电针组和假针组的术后咳嗽疼痛评分为 3.0±1.7

和4.4±1.7。在把握度为80%，双侧显著性水平为5%的情况下，每组需要24名患者。考虑到20%的脱落率，共需要60名患者(每组30名)。该预试验旨在为主要结局的收集初始数据，其结果将用于计算下一个更大的随机对照试验的样本量。

统计方法处理

采用 SPSS 27 软件进行统计分析，计量资料采用均数±标准差（M±SD）或中位数和四分位数间距表示，计数资料采用频数、构成比、百分比表示。计量资料的组间比较，采用独立样本的t检验或秩和检验；计数资料的组间比较采用 X^2 检验或秩和检验。检验水准为 0.05，即 P＜0.05 被认为所检验的差别有统计学意义。

对所有经随机化分组的病例，纳入治疗意向性分析（intention-to-treat analysis, ITT）；对主要结局同时采用符合方案数据分析（per-protocol analysis，PP）作为敏感性分析，纳入完成全部治疗的 80%，并无重大方案偏差或缺失的参与者；对缺失的数据，采用多重插补进行填补。对主要结局同时采用符合方案数据分析，作为敏感性分析。符合正态分布的计量资料采用t检验，不符合正态分布的计量资料采用秩和检验，计数资料采用 X^2 检验或非参数检验。我们将根据胸腔引流管放置时间、手术切口数量等对主要终点进行预定义的亚组分析。

病例的脱落与处理

脱落标准：受试者因为某些原因虽然通过知情同意、并且筛选合格获得随机号，但是不能完成研究方案所规定的疗程及观察周期，作为脱落病例。

脱落病例的处理：当受试者脱落后，研究者需采用登门、预约随访、电话、信件尽可能的与受试者联系，询问理由，记录最后一次针刺时间、完成所能完成的评估项目。脱落病例均应妥善保存有关试验资料，既作留档，也是进行意向性分析统计处理所需要的。脱落病人无需另补。

退出标准

受试者提出退出：

①疗效不佳；

②不能耐受不良反应；

③希望采取其他治疗方法；

④无任何理由主动退出。

终止标准

① 患者出现严重的不良反应，根据医生的判断需停止该病例临床试验。

② 出现其他影响试验观察的病证，根据医生判断应该停止临床试验者，作无效病例处理。

③ 临床试验方案实施中发生了重要偏差，如依从性太差等，难以评价针灸疗效。

④ 受试者在临床试验过程中不愿意继续进行临床试验，向主管医生提出退出临床试验的要求者。

剔除标准

①受试者出现违反纳排标准进入试验；

②违反方案规定的合并用药；

③错误的治疗分组；

④受试者用药依从性非常差。

质量控制

① 严格按照诊断、纳入和排除标准进行受试者的纳入。

② 随机号由专门独立于试验外的随机号管理员负责统一管理，只有在获得合格受试者具体信息并记录留档后才给予随机号，保障分配隐藏，防止随机号的提前泄露或漂移。

③ 经专家讨论，试验各个环节制定相关标准操作流程，使得各环节的操作有统一的标准，意见不一致时有依据可循。

④ 研究人员必须经过统一培训。内容包括熟悉本研究的目标、要求，掌握有关诊疗标准、随机分配方法、针刺的操作方法、测评表的使用，并对不同分工的研究者设立不同的培训时长，研究中心的相同分工的研究者需要通过一致性检验才能开展试验，要求一致性检验使用视频资料留档保存。

⑤研究分中心监查人员每月 2 次对完成的病例进行监查，指出其中存在的问题，并指导其改正。

⑥研究总中心监查人员审核试验流程，考察试验中涉及的伦理问题，如不

符合伦理原则，责令其整改。定期、不定期监查试验进程，指出其中存在的问题，并指导其改正。

风险及处理

针刺风险：

针刺过程中随时进行不良反应评价包括血肿、晕针、针刺后遗感等情况。

晕针处理措施：

停止针刺，将针全部起出，嘱患者平卧，注意保暖。

血肿处理措施：

微量皮下出血而致小块青紫时, 一般不需处理, 可自行消退。局部肿胀疼痛较剧、青紫面积较大时, 冷敷止血。

针刺后遗感处理措施：

轻的用手指在局部上下巡按，即可消失或改善；重的除在局部上下巡按外，并可用艾条施灸，也可很快消除。

总结与资料保存

临床观察完成后，资料总结由本课题管理办公室负责总结，所有资料原件经分中心负责人审核签字，科研部门盖章后保存于课题承担单位的科研资料档案室。
