# Supplementary material for: Electroacupuncture for acute postoperative pain during coughing after video-assisted thoracoscopic surgery: study protocol for a pilot randomized controlled trial
Source: PLoS One. 2025 Mar 26;20(3):e0316698. doi: 10.1371/journal.pone.0316698 (PMC11940426; doi:10.1371/journal.pone.0316698)
Supplement: Attachment — Submitted filename: Response to Reviewers.pdf [file pone.0316698.s005.pdf]

Dear editor,

Thank you very much for giving us the opportunity to revise our manuscript. We are grateful to the editor and reviewers for their positive and constructive comments and suggestions on our manuscript entitled “Electroacupuncture for acute postoperative pain after video-assisted thoracoscopic surgery: study protocol for a pilot randomized controlled trial” (PONE-D-24-42025), which certainly help us to improve it.

A point-by-point response to the editor’s and reviewers’ comments is attached, and the revision has been marked in red in the manuscript. We hope that these revisions successfully address their concerns and requirements.

Looking forward to hearing from you soon.

Sincerely,

Jian-Feng Tu, M.D.,
School of Acupuncture-Moxibustion and Tuina
Beijing University of Chinese Medicine
11 North Third Ring Road East, Chaoyang District
Beijing, China 100029
Email: tujianfeng1@126.com

Journal requirements:

- Please ensure that your manuscript meets PLOS ONE's style requirements, including those for file naming. The PLOS ONE style templates can be found at https://journals.plos.org/plosone/s/file?id=wjVg/PLOSOne_formatting_sample_main_body.pdf and https://journals.plos.org/plosone/s/file?id=ba62/PLOSOne_formatting_sample_title_authors_affiliations.pdf

Response: Thank you for your suggestions. In the resubmitted manuscript, we have improved the manuscript and file naming to meet PLOS ONE's style requirements.

-We note that the grant information you provided in the ‘Funding Information’ and ‘Financial Disclosure’ sections do not match. When you resubmit, please ensure that you provide the correct grant numbers for the awards you received for your study in the ‘Funding Information’ section.

Response: Sorry. This study is supported by the Qihuang Scholar Project of the National Administration of Traditional Chinese Medicine. We have updated statement on financial disclosure in the cover letter. Besides, this fund has no grant numbers.

-We note that you have indicated that there are restrictions to data sharing for this study. For studies involving human research participant data or other sensitive data, we encourage authors to share de-identified or anonymized data. However, when data cannot be publicly shared for ethical reasons, we allow authors to make their data sets

available upon request. For information on unacceptable data access restrictions, please see http://journals.plos.org/plosone/s/data-availability#loc-unacceptable-data-access-restrictions.

Before we proceed with your manuscript, please address the following prompts:

a) If there are ethical or legal restrictions on sharing a de-identified data set, please explain them in detail (e.g., data contain potentially identifying or sensitive patient information, data are owned by a third-party organization, etc.) and who has imposed them (e.g., a Research Ethics Committee or Institutional Review Board, etc.). Please also provide contact information for a data access committee, ethics committee, or other institutional body to which data requests may be sent.

b) If there are no restrictions, please upload the minimal anonymized data set necessary to replicate your study findings to a stable, public repository and provide us with the relevant URLs, DOIs, or accession numbers. Please see http://www.bmj.com/content/340/bmj.c181.long for guidelines on how to de-identify and prepare clinical data for publication. For a list of recommended repositories, please see https://journals.plos.org/plosone/s/recommended-repositories. You also have the option of uploading the data as Supporting Information files, but we would recommend depositing data directly to a data repository if possible.

Please update your Data Availability statement in the submission form accordingly.

Response: Thank you for your kind suggestions. There are no restrictions to data sharing for this study. If reviewers or readers have any questions regarding our

published data, they can contact the corresponding authors to access the original data. We have updated my Data Availability statement in the manuscript as “It is permitted for reviewers or readers to access the original data by contacting the corresponding author (Jian-Feng Tu)” (at Lines 414-415).

-We note that Figure 4 in your submission contain copyrighted images. All PLOS content is published under the Creative Commons Attribution License (CC BY 4.0), which means that the manuscript, images, and Supporting Information files will be freely available online, and any third party is permitted to access, download, copy, distribute, and use these materials in any way, even commercially, with proper attribution. For more information, see our copyright guidelines: http://journals.plos.org/plosone/s/licenses-and-copyright.

We require you to either (1) present written permission from the copyright holder to publish these figures specifically under the CC BY 4.0 license, or (2) remove the figures from your submission:

a. You may seek permission from the original copyright holder of Figure 4 to publish the content specifically under the CC BY 4.0 license.

We recommend that you contact the original copyright holder with the Content Permission Form (http://journals.plos.org/plosone/s/file?id=7c09/content-permission-form.pdf) and the following text:

“I request permission for the open-access journal PLOS ONE to publish XXX under

the Creative Commons Attribution License (CCAL) CC BY 4.0 (http://creativecommons.org/licenses/by/4.0/). Please be aware that this license allows unrestricted use and distribution, even commercially, by third parties. Please reply and provide explicit written permission to publish XXX under a CC BY license and complete the attached form."

Please upload the completed Content Permission Form or other proof of granted permissions as an "Other" file with your submission.

In the figure caption of the copyrighted figure, please include the following text: "Reprinted from [ref] under a CC BY license, with permission from [name of publisher], original copyright [original copyright year]."

b. If you are unable to obtain permission from the original copyright holder to publish these figures under the CC BY 4.0 license or if the copyright holder's requirements are incompatible with the CC BY 4.0 license, please either i) remove the figure or ii) supply a replacement figure that complies with the CC BY 4.0 license. Please check copyright information on all replacement figures and update the figure caption with source information. If applicable, please specify in the figure caption text when a figure is similar but not identical to the original image and is therefore for illustrative purposes only.

Response: Thank you for your kind suggestions. We have supplied a replacement figure and gotten permission from the copyright holder to publish this figure. The completed Content Permission Form has been uploaded as an "Other" file. In the caption of figure 4, we have included the following text: "Reprinted from [3Dbody

application] under a CC BY license, with permission from [3Dbody (Shanghai) Digital Technology Co., Ltd.], original copyright [2024].”

-Please review your reference list to ensure that it is complete and correct. If you have cited papers that have been retracted, please include the rationale for doing so in the manuscript text, or remove these references and replace them with relevant current references. Any changes to the reference list should be mentioned in the rebuttal letter that accompanies your revised manuscript. If you need to cite a retracted article, indicate the article’s retracted status in the References list and also include a citation and full reference for the retraction notice.

Response: Thank you for your suggestions. There are no retracted articles in the reference list. However, we found that some cited articles lacked information. We have modified the 9th, 21st, and 22nd references.

Reviewer: 1

As the authors calculated a sample size and defined a primary outcome to conduct a formal statistical analysis to evaluate effect size of the intervention, it is not a pilot study. It's not a pilot study simply because the sample size is small.

Response: Thank you for your valuable comments. Your perspective aligns with many scholars. However, it is indeed a topic of debate whether to calculate the sample size in the pilot study. Several scholars think the justification of the sample size for a pilot study is necessary and the primary outcome should be defined in the pilot study [1-3]. The sample size has been calculated in some published pilot studies [4,5]. Therefore, we cursorily calculated the sample size for this study. We are able to obtain relatively stable data from the pilot study, which is crucial for preventing under powering in future full-scale trial.

References

1. Kunselman AR. A brief overview of pilot studies and their sample size justification. Fertility and sterility. 2024;121(6):899-901. https://doi.org/10.1016/j.fertnstert.2024.01.040 PMID: 38331310

2. Fayers PM, Machin D. Sample size: how many patients are necessary? British journal of cancer. 1995;72(1):1-9. https://doi.org/10.1038/bjc.1995.268 PMID: 7599035

3. Wang Y, Wang LQ, Yang JW, Shi GX, Tu JF, Yan SY, et al. Application and reflection on pilot study in acupuncture clinical research. Zhongguo Zhen Jiu.

2021;41(3):325-9. https://doi.org/10.13703/j.0255-2930.20201024-0002 PMID: 33798319.

4. Lin LL, Tu JF, Wang LQ, Yang JW, Shi GX, Li JL, et al. Acupuncture of different treatment frequencies in knee osteoarthritis: a pilot randomised controlled trial. Pain. 2020;161(11):2532-8. https://doi.org/10.1097/j.pain.0000000000001940 PMID: 32483056

5. Swartz HA, Rollman BL, Mohr DC, Sadow S, Frank E. A randomized pilot study of Rhythms And You (RAY): An internet-based program for bipolar disorder administered with and without clinical helper support in primary care. Journal of affective disorders. 2021;295:183-91. https://doi.org/10.1016/j.jad.2021.08.025

-Since it is a sham-controlled trial protocol, I would like the authors to report on detailed wording given to participants regarding sham. Were the participants aware of the existence of sham electroacupuncture and that they may be randomly allocated to either real or fake intervention? This information about whether the term 'fake', 'placebo', 'dummy' or 'sham' was used in the informed consent process is important in the aspects of participant blinding and the direction of trial outcomes.

Response: Thank you for your suggestions. After reviewing the checklist, we discovered an issue with the version of the uploaded informed consent. We apologize for our mistake and have resubmitted the informed consent. The above words are not mentioned in the informed consent but are replaced by micro-electroacupuncture to

maximize the effectiveness of blinding. The partial disclosure is often used in the study of acupuncture [1]. Sufficient blinding will increase the credibility of the measured outcomes [2]. We will report the blind index to assess the adequacy of blinding.

References

1. Foster NE, Thomas E, Barlas P, Hill JC, Young J, Mason E, et al. Acupuncture as an adjunct to exercise based physiotherapy for osteoarthritis of the knee: randomised controlled trial. BMJ (Clinical research ed). 2007;335(7617):436. https://doi.org/10.1136/bmj.39280.509803.BE PMID: 17699546

2. Lee YS, Kim SY, Lee H, Chae Y, Lee MS. ACURATE: A guide for reporting sham controls in trials using acupuncture. Journal of evidence-based medicine. 2023;16(1):82-90. https://doi.org/10.1111/jebm.12524 PMID: 36959765

-As per author guidelines of this journal, authors of protocols without data are strongly encouraged to state how they plan to share their data when it is completed or published. Stating 'No datasets were generated or analysed during the current study. All relevant data from this study will be made available upon study completion' does not give any information how data will be shared. Please state your plan how to share your data. Are your going to use publicly accessable link, or provide supplementary files of raw data/analytic codes?

Response: Thank you for your valuable suggestions. We apologize for our negligence. We have stated the plan for sharing data at Lines 414-415 in the manuscript as "It is

permitted for reviewers or readers to access the original data by contacting the corresponding author (Jian-Feng Tu)".

-I would like to reconsider "average" postoperative pain as a primary outcome. Usually pain intensity data measured with NRS or VAS are not normally distributed. It means that it is common that you get (almost) complete pain relief or (almost) no help at all. It is highly likely that you belong to either end of the NRS, good or bad. So if you use the "mean" data, you are using nobody's result. So in acute pain trials, it is appropriate to use responder/non-responder analysis and I recommend you reconsider adopting this approach and recalculating the sample size accordingly.

Response: Thank you for your suggestions. The primary outcome in this study is the global pain state during 72h, which is calculated by the average of the NRS scores at 24h, 48h, and 72h. This similar primary outcome is also used in other acute pain studies [1-3]. According to your opinion, we have added the response as the secondary outcome as follows "Besides, the responder rate will be measured at 72h; a responder is defined as a participant with an NRS score ≤ 4 when coughing" (at Lines 264-266). The NRS score of 4 is the threshold for tolerable postoperative pain [4].

References

1. Ilfeld BM, Plunkett A, Vijjeswarapu AM, Hackworth R, Dhanjal S, Turan A, et al. Percutaneous Peripheral Nerve Stimulation (Neuromodulation) for Postoperative Pain: A Randomized, Sham-controlled Pilot Study.

Anesthesiology. 2021;135(1):95-110. https://doi.org/10.1097/ALN.0000000000003776 PMID: 33856424

2. Jiwnani S, Ranganathan P, Patil V, Agarwal V, Karimundackal G, Pramesh CS. Pain after posterolateral versus nerve-sparing thoracotomy: A randomized trial. J Thorac Cardiovasc Surg. 2019; 157:380-6. https://doi.org/10.1016/j.jtcvs.2018.07.033 PMID: 30195601

3. Machado LA, Maher CG, Herbert RD, Clare H, McAuley JH. The effectiveness of the McKenzie method in addition to first-line care for acute low back pain: a randomized controlled trial. BMC medicine. 2010;8:10. https://doi.org/10.1186/1741-7015-8-10 PMID: 20102596

4. Gerbershagen HJ, Rothaug J, Kalkman CJ, Meissner W. Determination of moderate-to-severe postoperative pain on the numeric rating scale: a cut-off point analysis applying four different methods. British journal of anaesthesia. 2011;107(4):619-26. https://doi.org/10.1093/bja/aer195 PMID: 21724620.

-Please report datails about how you will assess participant blinding. Why are you going to evaluate blinding after the ‘2nd’ treatment, not after 1st or at the end of treatment? What is your rationale for selecting ‘after the 2nd treatment’? How many options are your going to give? Real or sham? Or Real, sham, or don’t know? Are you going to calculate blinding index and what statistical method are you going to use? All these information will be useful and you’ve got a plenty of space for such information as this is a protocol paper.

Response: Thank you for your kind advices. Evaluation of blindness after the first treatment will not predict the integrity of blinding in subsequent phase of the trial [1]. If blinding is evaluated at the end of treatment, participants may judge the grouping according to the clinical effectiveness [2]. Therefore, we evaluate blinding after the ‘2nd’ treatment. We have added the sentence at Lines 353-354 in the manuscript as “The James' blinding indices will be used to assess the adequacy of blinding.” We will give participants three options. A sentence has been added to the manuscript as “After the second EA/SA treatment, all patients will be asked to answer the question, ‘Which type of acupuncture do you think you received, EA or Micro-electroacupuncture or Unclear?’”. (Lines 297-300)

References

1. Hopton AK, Macpherson H. Assessing blinding in randomised controlled trials of acupuncture: challenges and recommendations. Chinese journal of integrative medicine. 2011;17(3):173-6. https://doi.org/10.1007/s11655-011-0663-9 PMID: 21359917

2. Sackett DL. Turning a blind eye: why we don't test for blindness at the end of our trials. BMJ (Clinical research ed). 2004;328(7448):1136. https://doi.org/10.1136/bmj.328.7448.1136-a PMID: 15130997

-Page 5, lines 79-81: "It is unclear that the efficacy of electroacupuncture in alleviating acute postoperative pain after VATS." It's grammatically incorrect.

Response: Thank you for your valuable suggestions. We have modified the sentence

at Lines 81-82 as "It is unclear whether EA can alleviate acute postoperative pain after VATS."

Reviewer: 2

-The primary outcome is the average postoperative pain score during coughing. Please revise the title to match the primary outcome.

Response: Thank you for your professional suggestions. We have revised the title to match the primary outcome as "Electroacupuncture for acute postoperative pain during coughing after video-assisted thoracoscopic surgery: study protocol for a pilot randomized controlled trial".

-Please provide the rationale for the treatment acupoints selection in the discussion section.

Response: Thank you for your comments. We have added the rationale for the treatment acupoints selection as "LR3, GB34, LU6, PC6, LI4, and Neimadian (extra points) have been used to reduce postoperative pain in patients undergoing thoracic surgery [14, 15, 23, 24]. According to the traditional Chinese medicine meridian theory and clinical experience, we finally determined the treatment acupoints for this study" at Line 377-381.

-The primary outcome is the average postoperative pain score during coughing, measured using a numeric rating scroe in the Abstract section(line 36-38, page 2). However, it is measured using the PCIA in the Outcomes section (line 242- 245). Which is correct?

Response: Thank you for your valuable suggestions. We apologize for our mistake. We have modified the sentence at Line 256 as “Pain scores will be measured using the NRS at 24, 48, and 72 h after surgery”.

-When is the evaluation time point for the primary endpoint? (at 24h or 48h or 72h after surgery) Please clarify it.

-Is the primary outcome the changes in NRS at each timepoint? Is the secondary outcome the changes in secondary outcome at each timepoint? Please clarify it.

Response: Thank you for your suggestions. The evaluation time point for the NRS is at 24h, 48h, and 72h after surgery. The primary outcome is the average of pain scores evaluated at 24h, 48h, and 72h after surgery. Pain score during coughing of NRS at each timepoint is a secondary outcome.
