# Supplementary material for: Electroacupuncture for acute postoperative pain during coughing after video-assisted thoracoscopic surgery: study protocol for a pilot randomized controlled trial
Source: PLoS One. 2025 Mar 26;20(3):e0316698. doi: 10.1371/journal.pone.0316698 (PMC11940426; doi:10.1371/journal.pone.0316698)
Supplement: Attachment — Submitted filename: Response_to_Reviewers_auresp_2.pdf [file pone.0316698.s006.pdf]

Dear editors,

Thank you for your letter and comments concerning our manuscript entitled "Electroacupuncture for acute postoperative pain during coughing after video-assisted thoracoscopic surgery: study protocol for a pilot randomized controlled trial" (PONE-D-24-42025_R1). Those positive and constructive comments and suggestions are valuable and helpful for revising and improving our paper.

A point-by-point response to the editor's comments is attached, and the revision has been marked in red in the manuscript. We hope that these revisions successfully address their concerns and requirements.

Looking forward to hearing from you soon.

Sincerely,

Jian-Feng Tu, M.D.,

School of Acupuncture-Moxibustion and Tuina

Beijing University of Chinese Medicine

11 North Third Ring Road East, Chaoyang District

Beijing, China 100029

Email: tujianfeng1@126.com

Journal requirements:

-Please review your reference list to ensure that it is complete and correct. If you have cited papers that have been retracted, please include the rationale for doing so in the manuscript text, or remove these references and replace them with relevant current references. Any changes to the reference list should be mentioned in the rebuttal letter that accompanies your revised manuscript. If you need to cite a retracted article, indicate the article's retracted status in the References list and also include a citation and full reference for the retraction notice.

Response: Thank you for your suggestions. We apologize for our mistake. Some references may be incomplete or incorrect, but no retracted paper in the reference list. Two papers (10 and 11) are not in English. For the convenience of readers, we have replaced them with relevant English papers. We have checked again and re-imported all the references.

Editor Comments:

-The article only briefly mentions the assessment of whether blinding was successful for the patients: "The James' blinding indices will be used to assess the adequacy of blinding." Please provide more specific relevant details and references of the James' blinding indices. Notably, the Bang's blinding index is also a commonly used method in the field of sham acupuncture to assess whether patient blinding was successful. Please clarify the differences between the two methods and explain why you ultimately chose the James' blinding indices over the Bang's blinding index.

Response: Thank you for your valuable comments. James' blinding index is different from Bang's blinding index. James' blinding index combines blinding data from all arms, but Bang's blinding index assesses blinding separately for different arms [1]. Therefore, we will use James' and Bang's blinding index to assess the adequacy of blinding (at Lines 356-357). We have added the sentence at Lines 298-303 in the manuscript: "The James' blinding index ranges from 0 to 1, whereby 0 corresponds to no blinding, 0.5 corresponds to completely random blinding, and 1 corresponds to complete blinding [19]. The Bang's blinding index ranges from -1 to 1, with 1 indicating complete lack of blinding, 0 indicating perfect blinding, and -1 indicating opposite guessing [20]."

References

1. Bang H, Ni L, Davis CE. Assessment of blinding in clinical trials. Controlled clinical trials. 2004;25(2):143-56. doi: 10.1016/j.cct.2003.10.016. PubMed PMID: 15020033.

-In the revised manuscript, "a responder is defined as a participant with an NRS score ≤ 4 when coughing" (at Lines 264-266). However, the baseline NRS score of patients will also impact the post-treatment score. Why not define a responder based on a certain threshold of change in NRS score before and after treatment?

Response: Thank you for your suggestions. The NRS was not evaluated before treatment in this study. Due to the varying efficacy of anesthetics on patients, the time for patients to be fully awake after surgery is different. If patients are not fully awake, we think that the NRS scores they give are inaccurate. Hence, we don't evaluate the NRS scores before treatment, which is similar to other studies [1-3].

References

1. Ibrahim M, Menna C, Andreetti C, Puyo C, Maurizi G, D'Andrilli A, et al. Does a Multimodal No-Compression Suture Technique of the Intercostal Space Reduce Chronic Postthoracotomy Pain? A Prospective Randomized Study. Journal of thoracic oncology. 2016;11(9):1460-8. doi: 10.1016/j.jtho.2016.05.023. PubMed PMID: 27282308.
2. Ding W, Chen Y, Li D, Wang L, Liu H, Wang H, et al. Investigation of single-dose thoracic paravertebral analgesia for postoperative pain control after thoracoscopic lobectomy - A randomized controlled trial. International journal of surgery. 2018;57:8-14. doi: 10.1016/j.ijsu.2018.07.006. PubMed PMID: 30056127.
3. Jiwnani S, Ranganathan P, Patil V, Agarwal V, Karimundackal G, Pramesh CS. Pain after posterolateral versus nerve-sparing thoracotomy: A randomized trial. The Journal of thoracic and cardiovascular surgery. 2019;157(1):380-6. doi:

10.1016/j.jtcvs.2018.07.033. PubMed PMID: 30195601.
