# Supplementary material for: Electroacupuncture for acute postoperative pain during coughing after video-assisted thoracoscopic surgery: study protocol for a pilot randomized controlled trial
Source: PLoS One. 2025 Mar 26;20(3):e0316698. doi: 10.1371/journal.pone.0316698 (PMC11940426; doi:10.1371/journal.pone.0316698)
Supplement: Attachment — Submitted filename: Response_to_Reviewers_auresp_3.pdf [file pone.0316698.s007.pdf]

Dear editors,

Thank you for your letter and comments concerning our manuscript entitled “Electroacupuncture for acute postoperative pain during coughing after video-assisted thoracoscopic surgery: study protocol for a pilot randomized controlled trial” (PONE-D-24-42025_R2). We are grateful to the editor and reviewers for their positive and constructive suggestions which certainly help us to improve it.

A point-by-point response to the editor’s comments is attached, and the revision has been marked in red in the manuscript. We hope that these revisions successfully address their concerns and requirements.

Looking forward to hearing from you soon.

Sincerely,

Jian-Feng Tu, M.D.,

School of Acupuncture-Moxibustion and Tuina

Beijing University of Chinese Medicine

11 North Third Ring Road East, Chaoyang District

Beijing, China 100029

Email: tujianfeng1@126.com

Journal requirements:

- Please review your reference list to ensure that it is complete and correct. If you have cited papers that have been retracted, please include the rationale for doing so in the manuscript text, or remove these references and replace them with relevant current references. Any changes to the reference list should be mentioned in the rebuttal letter that accompanies your revised manuscript. If you need to cite a retracted article, indicate the article's retracted status in the References list and also include a citation and full reference for the retraction notice.

Response: Thank you for your suggestions. We have thoroughly checked the reference list and no retracted articles have been cited.

Reviewer: 3

- The document is well written. There are a few data issues that should be clarified. For example, the quality control section appears to be directed to the researchers. The investigators state that the researcher will regularly monitor the trial. What exactly is, 'regularly'?

Response: Thank you for your suggestions. 'Regularly' means that we will conduct monthly monitoring of the trial. [1]. We have revised the sentence at Line 329 in the manuscript as "A unified CRF will be used for recording, and the researcher will monthly monitor the trial."

References

1. Wu XK, Gao JS, Ma HL, Wang Y, Zhang B, Liu ZL, et al. Acupuncture and

Doxylamine-Pyridoxine for Nausea and Vomiting in Pregnancy : A Randomized, Controlled, 2 × 2 Factorial Trial. Annals of internal medicine. 2023;176(7):922-33. doi: 10.7326/m22-2974. PubMed PMID: 37335994.

-Also in the pain assessment, the patient will rate their pain which can be somewhat subjective. How will this assessment be validated? This should at least be mentioned in the protocol.

Response: Thank you for your valuable comments. In our study, postoperative pain will be evaluated by the validated NRS [1]. Besides, the NRS has always been used in many high-quality clinical studies [2-4]. We have added this sentence to the manuscript (at Lines 257-258).

References

1. Ferreira-Valente MA, Pais-Ribeiro JL, Jensen MP. Validity of four pain intensity rating scales. Pain. 2011;152(10):2399-404. doi: 10.1016/j.pain.2011.07.005. PubMed PMID: 21856077.
2. Malfliet A, Kregel J, Coppieters I, De Pauw R, Meeus M, Roussel N, et al. Effect of Pain Neuroscience Education Combined With Cognition-Targeted Motor Control Training on Chronic Spinal Pain: A Randomized Clinical Trial. JAMA neurology. 2018;75(7):808-17. doi: 10.1001/jamaneurol.2018.0492. PubMed PMID: 29710099.
3. Tankha H, Gaskins D, Shallcross A, Rothberg M, Hu B, Guo N, et al. Effectiveness of Virtual Yoga for Chronic Low Back Pain: A Randomized Clinical Trial. JAMA network open. 2024;7(11):e2442339. doi: 10.1001/jamanetworkopen.2024.42339.

PubMed PMID: 39485352.
4. Bendixen M, Jørgensen OD, Kronborg C, Andersen C, Licht PB. Postoperative pain and quality of life after lobectomy via video-assisted thoracoscopic surgery or anterolateral thoracotomy for early stage lung cancer: a randomised controlled trial. The Lancet Oncology. 2016;17(6):836-44. doi: 10.1016/s1470-2045(16)00173-x. PubMed PMID: 27160473.

- Concerning the sample size and analysis, the sample size appears adequate for a pilot effort. For comparative univariate statistical analysis, the t-test and Wilcoxon appear to suffice as needed. Are there any thoughts of any adjustment (multivariate perhaps) to the analysis based on relevant patient characteristics or clinical variables?

Response: Thank you for your comments. We will use a linear regression model as the sensitivity analysis to examine the robustness of the result. Based on the previous research, we found that some factors impact the primary outcome such as sex, age, smoking history, analgesic consumption, chest tube duration, and the number of ports [1-3]. We have added the sentence at Lines 354-358 in the manuscript as "Besides, to examine the robustness of the result, the primary outcome will be performed using a linear regression model as sensitivity analysis adjusted for sex, age, smoking history at baseline, and analgesic consumption, chest tube duration, the number of ports."

References

1. Sun K, Liu D, Chen J, Yu S, Bai Y, Chen C, et al. Moderate-severe postoperative pain in patients undergoing video-assisted thoracoscopic surgery: A retrospective study.

Scientific reports. 2020;10(1):795. doi: 10.1038/s41598-020-57620-8. PubMed PMID: 31964955.

2. Yun T, Zhang Y, Liu A, Qin Y, Sun X, Wu Z, et al. Randomized Trial of Modified Chest Tube Placement vs Routine Placement After Lung Resection. The Annals of thoracic surgery. 2023;116(5):1013-9. doi: 10.1016/j.athoracsur.2023.04.031. PubMed PMID: 37146783.

3. Tamura M, Shimizu Y, Hashizume Y. Pain following thoracoscopic surgery: retrospective analysis between single-incision and three-port video-assisted thoracoscopic surgery. Journal of cardiothoracic surgery. 2013;8:153. doi: 10.1186/1749-8090-8-153. PubMed PMID: 23759173.

-Also, presumably statements such as," We will perform a predefined subgroup analysis of primary endpoints according to chest tube duration and the number of incisions.", will be detailed in the protocol.

Response: Thank you for your valuable suggestions. Previous studies found that the average chest tube duration was about 3 days and the number of ports affected postoperative pain [1-2]. We have revised the sentence at Lines 358-360 in the manuscript as "We will perform a predefined subgroup analysis of primary outcome according to chest tube duration (< 3/≥3 days), and the number of ports (1/2/3).

References

1. Yun T, Zhang Y, Liu A, Qin Y, Sun X, Wu Z, et al. Randomized Trial of Modified Chest Tube Placement vs Routine Placement After Lung Resection. The Annals of

thoracic surgery. 2023;116(5):1013-9. doi: 10.1016/j.athoracsur.2023.04.031. PubMed PMID: 37146783.

2. Tamura M, Shimizu Y, Hashizume Y. Pain following thoracoscopic surgery: retrospective analysis between single-incision and three-port video-assisted thoracoscopic surgery. Journal of cardiothoracic surgery. 2013;8:153. doi: 10.1186/1749-8090-8-153. PubMed PMID: 23759173.
